# 

**Published:** 1879-12

**Authors:** 


CONTENTS.

| | PAGE |
|---|---|
| INTRODUCTORY | 1 |
| ANNUAL AILMENTS | 2 |
| CONSUMPTION | 6 |
| PRACTICAL KNOWLEDGE | 7 |
| A COMPLIMENT TO THE PHYSICIAN | 8 |
| MARVELS OF MAN | 9 |
| RECREATION | 11 |
| SMALL-POX | 12 |
| THE TEETH | 14 |
| SLEEPING ROOMS | 16 |
| FARMERS AND CITIZENS | 17 |
| OVER-EATING | 19 |
| GOING IT | 21 |
| A DANGEROUS CURIOSITY | 22 |
| THE FEET IN WINTER | 23 |
| BATHS AND BATHING | 24 |
| BARE NECK AND ARMS | 25 |
| SPRAINS | 26 |
| HEART AFFECTIONS | 27 |
| OUT-DOOR SAFETY | 28 |
| HABIT | 29 |
| SLEEPING POSITION | 30 |
| HEALTH | 31 |
| A NATURAL WISH | 31 |

